# Narrative Skills in Primary School Children with Autism in Relation to Language and Nonverbal Temporal Sequencing

**DOI:** 10.1007/s10936-020-09703-w

**Published:** 2020-04-13

**Authors:** Emilia Carlsson, Jakob Åsberg Johnels, Christopher Gillberg, Carmela Miniscalco

**Affiliations:** 1 Speech and Language Pathology Unit, Institute of Neuroscience and Physiology, University of Gothenburg, Box 452, 405 30 Gothenburg, Sweden; 2 Gillberg Neuropsychiatry Centre, Institute of Neuroscience and Physiology, University of Gothenburg, Gothenburg, Sweden; 3 Department of Child Neuropsychiatry, Queen Silvia Children’s Hospital, Sahlgrenska University Hospital, Gothenburg, Sweden; 4 Department of Paediatric Speech and Language Pathology, Queen Silvia Children’s Hospital, Gothenburg, Sweden

**Keywords:** Narrative skills, Autism, Children, Language skills, Nonverbal temporal sequencing

## Abstract

Recent research has suggested that temporal sequencing of narrative events might be a domain-general ability that underlies oral narrative capacities. The current study investigated this issue in a group of children with known pragmatic and narrative difficulties, namely Autism Spectrum Disorder (ASD). We hypothesized (1) that children with ASD (n = 45) would retell narratives of poorer quality than both chronological age-matched (CAM) children and younger children matched on sentence-level language skills (LM), and (2) that nonverbal temporal sequencing skills would uniquely predict individual differences in oral narrative performance in children with ASD. The results show that children with ASD performed poorer on all measures of oral narrative quality compared with the CAM group, and on eight of ten measures compared with the LM group. Thus, our first hypothesis was confirmed, suggesting that narrative difficulties in ASD cannot be fully explained by impaired language. The second hypothesis was only partly confirmed: nonverbal temporal sequencing explained significant or marginally significant variance in some, but not all, aspects of oral narrative performance of children with ASD. These results are discussed from theoretical and clinical/educational perspectives, in relation to the heterogeneity of language skills in ASD and to domain-general features of narrative processing.

## Introduction

Storytelling was widespread long before literacy emerged. Narrative ability reflects our ability to dress our thoughts and experiences in words and to convey events using language in communicative situations (Bruner 1986). Narrative development starts early in life and is entangled with cognition, social development, linguistic skills and world knowledge (Leinonen et al. 2000). Narrative capacity has implications for many aspects of children’s development, such as planning, organizing and sequencing one’s thoughts and developing a sense of identity (Berman 2009). Moreover, narrative ability is considered an ecologically valid way of capturing communicative competence in childhood (Botting 2002). Indeed, narrative ability has shown to predict future communicative functioning and persistent language impairment (Bishop and Edmundson 1987; Norbury and Bishop 2003), social interaction (Pelletier and Wilde Astington 2004), literacy and reading development (Cain and Oakhill 1996; Stothard et al. 1998) as well as future academic achievement (Fazio et al. 1996). In addition, there is a close link between narrative and general pragmatic ability (Reuterskiöld Wagner 1999). Thus, narrative capacities are considered to be an important skill to assess in individuals who experience pragmatic language difficulties, such as children with autism spectrum disorder (ASD) (APA 2013; Baixauli et al. 2016; Bruner and Feldman 1993; Miniscalco et al. 2007). Many children with ASD struggle with narrative performance (e.g. Baixauli et al. 2016), and a substantial proportion also struggle with language processing at the level of words and single sentences (e.g. Eigisti et al. 2011; Tager-Flusberg and Joseph 2003). Currently, it is not entirely clear whether difficulties with narrating are accounted for by such limitations in language skills at the level of single sentences (henceforth *language skills*) or if other factors are also involved.

It is methodologically challenging to determine if oral narrative difficulties in children with ASD is more severe than expected based on their language skills. One procedure has been to compare ASD children with language-matched comparison groups. There has, however, been concerns raised that tight group matching can jeopardize the representativeness of the study samples since children with ASD and typically developing children typically differ in their language skills (Charman 2004). In order to better maintain representativeness, sometimes older children with ASD are matched with younger typically developing children at the same language level. In a study by Diehl et al. (2006), when children with and without ASD were matched carefully on age, cognitive abilities, expressive and receptive language abilities children with ASD showed significant impairments in story coherence but not in story length or syntactic complexity (subordinate clauses) (Diehl et al. 2006). In another important study, children with ASD created narratives with shorter and less syntactic complex sentences than younger language matched non-ASD children (King et al. 2013). A study by Peristeri et al. (2017) compared children with ASD with high language level (HL), low language level (LL) and non-ASD children matched on language, age and cognitive abilities. The results showed that the narratives produced by children with ASD LL had lower syntactic complexity (fewer subordinate clauses) than the other two groups, while there was no difference between ASD HL and the non-ASD children on the same measure. In the present work, we wish to contribute to the present knowledge on the role of language skills in narrative performance by examining narrative skills in a population-screened sample of children with ASD and compare with two comparison groups: carefully matched on language skills and on age.

**Fig. 1:**
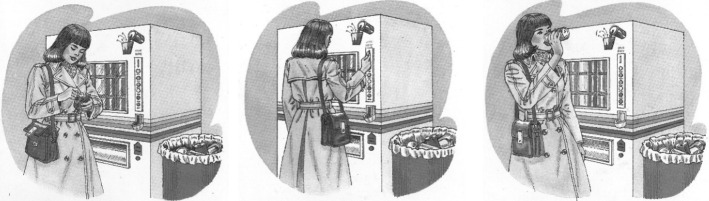
A set of pictures from the picture arrangement subtest in WISC-III. Printed with permission from Pearson Sweden

If, as we hypothesize, the narrative difficulties in ASD cannot be fully explained by concurrent language difficulties, then additional factors might be involved. One specific skill of interest in the present paper is nonverbal temporal sequencing. This focus is motivated by recent research suggesting a parallel sequential organisation between oral narrative sequential processing in the oral and visual domains (e.g. Coderre et al. 2018; Cohn 2019). In a previous study, Åsberg Johnels et al. (2013), they examined narrative ability in children with neurodevelopmental disorders and its relation to language skills and to nonverbal temporal sequencing assessed using the Picture Arrangement Task from WISC III (Wechsler 1999). In the picture arrangement test, the test leader instructs the child to arrange a set of coloured pictures in the right order to produce a comprehensible story without any demands on spoken output (see Fig. 1). Regression analysis suggested that temporal sequencing, using this test constrained the ability to convey story information during oral narration independently of language capacity, was important for conveying story information (Åsberg Johnels et al. 2013). This finding, if replicated, has important clinical/educational and theoretical implications by pointing to the modality independent nature of narrative processing difficulties. Indeed, from a practical point of view, Coderre (2019) dismantled the “Visual Ease Assumption”, i.e. that visually presented materials is easier to understand than verbally presented materials in clinical populations, including children with ASD (Coderre 2019).

Relationships between nonverbal temporal sequencing, listening comprehension and language development have previously been demonstrated in children without ASD (Zampini et al. 2017). In the context of ASD, Coderre et al. (2018) compared visual and linguistic narrative processing in individuals with ASD and demonstrated similar difficulties in both modalities, suggesting a domain-general impairment in narrative comprehension (Coderre et al. 2018). In a similar vein and specifically with regard to narrative production, Åsberg Johnels (2018) speculated that temporal sequencing might be a domain-general ability underlying narrative difficulties, as expressed with pictures or with words. Perhaps language skills and nonverbal temporal sequencing can be said to constitute proximal building blocks of narrative performance (‘the simple view of narrating’) (Åsberg Johnels 2018) in much the same way as decoding and linguistic comprehension are said to be in reading (‘the simple view of reading’).

However, there are several limitations in prior research that make such assertions tentative. For instance, Coderre examined narrative comprehension, not production, in ASD, whereas Åsberg Johnels et al. (2013) used a mixed clinical sample, and not just children with ASD, in their study. Moreover, no comparison groups matched by age or by language level were included in the latter study. Consequently, we find it important to replicate and extend previous research using a clinical sample of children with ASD and two non-clinical comparison groups matched for age and language, respectively. The aim of the present study was to examine narrative ability in children with and without ASD, and to investigate to what extent narrative difficulties in children with ASD reflect difficulties with language and/or nonverbal temporal sequencing.

Our hypotheses were that

1. children with ASD would perform worse on the narrative task compared with both age- matched (CAM) and younger language-matched (LM) children.
2. nonverbal temporal sequencing predicts narrative performance in children with ASD independently of language skills.

## Methods

### Participants

In total, 45 children (8 girls, 37 boys) ages 5.9–9.8 (mean 7.6) years with ASD were included in this study. Of these children, 42 (93%) attended mainstream primary schools, one went to a special needs comprehensive school and two were in preschool. The participating children were recruited from a population-based sample (the AUDIE project) of N = 129 children who had screened positive for ASD at age 2.5 years in connection with their child health care centre’s routine check-up. The parents of 107 children agreed to have their child participate in the study (Kantzer et al. 2013, 2018). Of these, 85 children underwent a third follow-up assessment at the child neuropsychiatric clinic (CNC) in Gothenburg approximately 5 years after the first assessment. For various reasons, some of the 85 children were not included in the present study. The attrition included one group of children who either were unable to participate in a formal language assessment (*n* = 25) or did not produce the required number of five sentences in the BST assessment (Renfrew 1997; Svensson and Tuominen-Eriksson 2002) (*n* = 2). Another two children did not have a result for Test on Reception Of Grammar-2 (TROG-2) (Bishop 2003, Swedish version 2009) and were therefore excluded from further analysis, and 11 children were excluded because they did not meet all criteria for an ASD diagnosis according to clinical consensus based on all available information from all professionals involved.

#### Age- and Language Matched Groups

Forty-seven children without ASD (17 girls, 30 boys) ages 6.5–9.0 (mean 7.8) years were recruited from mainstream primary schools in western Sweden, matched by chronological age (CAM). The language-matched (LM) group consisted of 27 children without ASD (18 girls, 9 boys) ages 3.9–8.6 (mean 6.1) years. They were recruited in the same way as the CAM group; see Table 1. The children in the LM group were matched according to raw score results on TROG-2 (Bishop 2003). Those with a standard score of 70 or above were included; six children were excluded.

**Table 1:** Descriptive data and group comparisons for the tests in the three groups

Results at the school-year follow up	Mean (SD)			Group comparison
	ASD (*n* = 45)	CAM (*n* = 47)	LM (*n* = 27)	
Age (years)	7.6 (1.0)	7.8 (0.6)	6.1 (1.3)	ASD = CAM > LM
Language skills				
TROG-2 raw scores	9.0 (5.2)	14.8 (2.7)	9.7 (4.8)	ASD = LM < CAM
TROG-2 standard scores^c^	75.8 (21.0)	99.9 (12.7)	92.7 (15.2)	ASD < LM < CAM
Recalling sentences raw scores	22.1 (14.5)	40.2 (7.8)	28.1^a^ (12.0)	ASD = LM < CAM
Recalling sentences scaled scores^d^	6.9 (4.8)	13.9 (2.8)	12.0^b^ (3.6)	ASD < CAM = LM
The bus story test				
Information	21.4 (12.6)	33.8 (8.1)	22.4 (11.1)	ASD = LM < CAM
Sentence length	7.0 (1.8)	11.2 (2.1)	8.8 (2.3)	ASD < LM < CAM
Subordinate clauses	2.0 (1.9)	5.6 (2.7)	3.6 (2.5)	ASD < LM < CAM
Nonverbal cognitive ability^e^ raw scores	12.2 (6.3)	n.p	n.p	
Nonverbal cognitive ability T-scores	48.4 (9.9)	n.p	n.p	
Nonverbal temporal sequencing raw scores	14.3 (8.9)	n.p	n.p	
Nonverbal temporal sequencing scaled scores	8.4 (3.5)	n.p	n.p	

*ASD* autism spectrum disorder, *CAM* Chronological age-matched; *LM* language-matched

^a^*n* = 26

^b^*n* = 21

^c^(M = 100, SD = 15)

^d^(M = 10, SD = 3)

^e^Matrix reasoning and Nonverbal sequential reasoning (*n* = 44)

Significant differences were found when the groups were compared on raw scores on TROG-2: H (119) = 32.86, *p* < 0.01, and Recalling Sentences (CELF-4): H (118) = 44.10, *p* < 0.01. No differences between the ASD and LM group were found on raw scores on TROG-2 (*p* = 1.0) or Recalling Sentences (*p* = 0.255) (see Table 1). The ASD group was outperformed by the age-matched children on both tests (both *p* < 0.001). The CAM group had higher raw scores on both tests (*p* < 0.001) compared with the LM group, reflecting the fact that typically developing children score higher with increasing age. Significant differences across the three groups were found on age-adjusted scores of receptive grammar (TROG-2, standard scores) (H[119] = 30.86, *p* < 0.01) and Recalling Sentences (scaled scores) (H [113] = 44.01 *p* < 0.01). The LM group and the CAM group did not differ significantly on TROG-2 standard scores (*p* = 0.197) or on Recalling Sentences scaled scores (*p* = 0.352). By contrast, the ASD group was outperformed by both groups on TROG-2 (LM *p* = 0.01; CAM* p* =  < 0.001) and Recalling Sentences (both *p* =  < 0.001). There was no difference in age between the ASD group and the CAM group (*p* = 1.0).

### Procedure

Two speech and language pathologists (SLPs) at the CNC assessed all children with ASD in connection with the third follow-up assessment, during 1–2 visits to the clinic. Each visit lasted about 60 min. The first author and two SLP master’s students assessed the comparison groups for approximately 45–60 min, with breaks taken if needed, in a separate, quiet room at their schools.

### Material

#### Narrative Ability

The Bus Story Test (BST) (Renfrew 1997, Swedish version, Svensson and Tuominen-Eriksson 2002) consists of a coloured picture storybook about a ‘naughty’ bus. The test leader reads the story and then the child is asked to retell the story while looking at the 12 pictures. All stories were audio recorded and orthographically transcribed according to the Swedish manual. The Information score (max = 54), Subordinate Clauses (i.e. number of produced subordinate clauses) within the retold story, and Sentence Length (i.e. number of words in the five longest sentences divided by five) were calculated. The test is standardized for Swedish children in the 3.9–8.5 year age range, which does not fully cover the age range in the present study. Consequently, the BST results are presented as raw scores. The *Narrative Analysis Profile* was used for further analysis of the children’s narrative ability on six dimensions: *Topic Maintenance*, *Event Sequencing, Explicitness, Referencing, Conjunctive Cohesion* and *Fluency* (Bliss et al. 1998). Each dimension results in a score from 1 to 3, where 1 = inappropriate, 2 = variable (a mix of appropriate and inappropriate behaviours) and 3 = appropriate. NAP total is a total score where the score of the six dimensions are added together (min 6–max 18).

#### Receptive and Expressive Language

Language comprehension (receptive grammar) was assessed with the TROG-2 (Bishop 2003*,* Swedish version 2009). In TROG-2, the task is to match orally presented sentences with the correct picture out of a choice of four. The results are presented in terms of both raw scores (number of correctly solved blocks out of a maximum of 20) and standard scores (M = 100, SD = 15) based on Swedish norms. The Cronbach’s alpha is 0.89 in the Swedish manual.

The Recalling Sentences subtest from the Clinical Evaluation of Language Fundamentals-4 (CELF-4; Semel et al. 2003, Swedish version 2013) was used as a measure of language production and expressive language (Klem et al. 2015). Recalling Sentences consists of 24 sentences. The participant is to repeat each sentence produced by the test leader, resulting in a score from 0 (> 4 errors) to 3 (no errors). The maximum score is 72. The results are presented in raw scores and scaled scores (around a normative M = 10, SD = 3; Swedish norms). The version of the Recalling Sentences subtest from CELF-4 used in this study was the version used in the standardization of the instrument rather than the final, published version, which includes some revisions of items. This makes the actual scaled score results a bit uncertain, and they should therefore be considered as rough estimates. However, it is important to note that both children with and without ASD were assessed using the same version and that the analyses conducted were based on raw scores.

#### Nonverbal Cognitive Ability in the ASD Group

Nonverbal cognitive ability was measured with the Matrix Reasoning subtest from the Wechsler Abbreviated Scales of Intelligence (Wechsler 1999) (for the children with ASD only). The results are presented as raw and *T-*scores (M** = **50, SD = 10) based on US norms; no Swedish norms are available.

#### Nonverbal Temporal Sequencing in the ASD Group

**Fig. 2:**
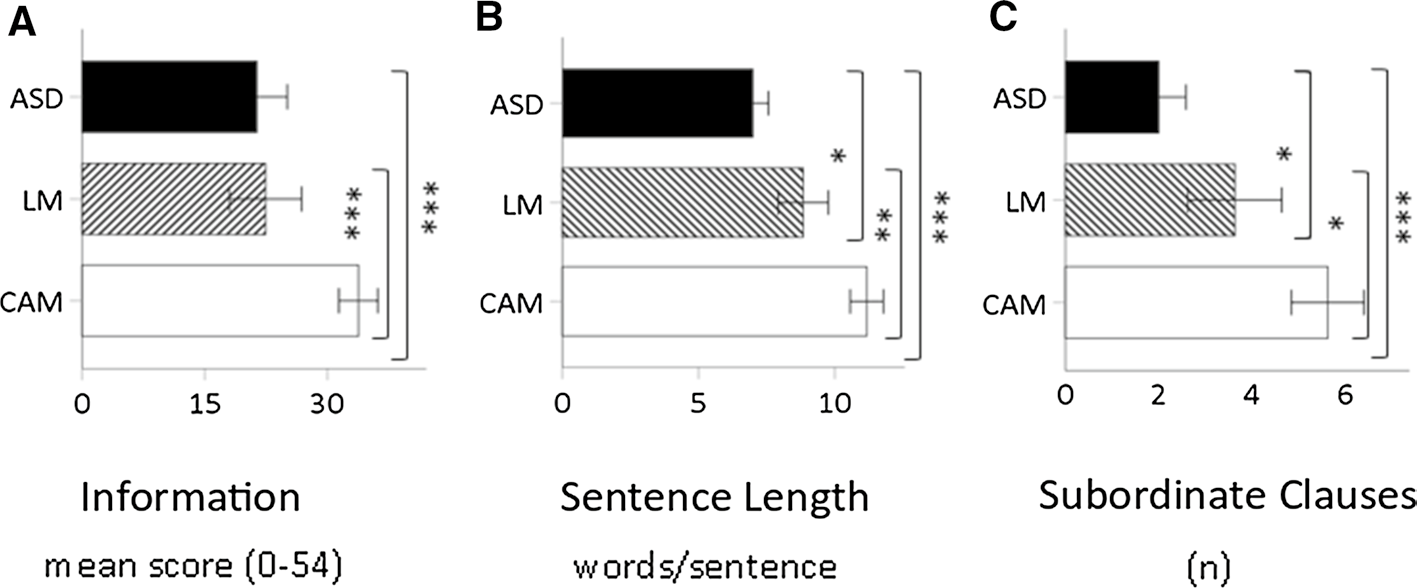
The three measures of narrative ability assessed using the Bus Story Test, compared between the three groups of children: *ASD* autism spectrum disorder (n = 45), *CAM* chronological-age-matched (n = 47), and *LM* language-matched (n = 27). Panel A: the bars represent the group mean of the information score, which ranged from 0 to 54. Panel B: the bars represent sentence length, i.e. the group mean of number of words/sentence. Panel C: the bars represent subordinate clauses, i.e. group mean of the number of subordinate clauses. Error bars show the 95% CI. *p < .05, **p < *.*01, ***p < .001

Nonverbal temporal sequencing was assessed using the Picture Arrangement subtest from WISC–III (Wechsler 1999) for the children with ASD only. The SLP instructed the child to arrange a set of coloured cartoon pictures (14 sets of 3–5 pictures) into a comprehensible story (see Fig. 2). The child scored 2 points for arranging a correct set within the assigned time (age norms) and 3 extra points for speed. After failing three sets, the test was ended. The results are expressed in scaled scores around the normative mean of 10 (SD ± 3).

#### Reliability

The first author calculated the three BST scores for the ASD group. Eleven (24%) of the BST transcriptions were then re-evaluated by a second independent experienced SLP. To calculate the reliability, intra-class correlation coefficient (ICC) was used (Fleiss 1986). The inter-rater reliability ranged from excellent to good for all three BST scores: Information (0.97), Sentence Length (0.91) and Subordinate Clauses (0.74) (two-way random, single measures). Twelve (27%) of the transcripts in the ASD group were re-evaluated by the first author, and the intra-rater reliability values were found to range from excellent to good for Information (0.98), Sentence Length (0.90) and Subordinate Clauses (0.78) (one-way random single measures). Then 14 (19%) of the transcripts from the comparison groups (LM and CAM combined) were re-evaluated (two-way random, single measures), and good reliability was found for Information (0.85), Sentence Length (0.89) and Subordinate Clauses (0.82). Overall, the reliability of the data coding appeared to be sufficient. The NAP analysis was performed on all BST transcriptions by the first author and 26 (22%) transcriptions of the material were then rated by an independent blinded SLP in order to calculate inter-rater reliability, which resulted in an ICC of 0.81 (two-way random, single measures).

### Ethics

The study received ethical approval from the Regional Ethical Review Board in Gothenburg*,* Sweden (case number 723-13). All parents of the participating children provided oral and written informed consent.

### Statistical Analyses

Non-parametric tests were used for group comparisons (a Kruskal–Wallis test with pairwise comparisons). The significance levels within the group comparisons were adjusted for multiple comparisons (Bonferroni). The correlations were calculated using Spearman’s rho correlation, and an alpha level was set at *p* < 0.05. Linear regression analyses were conducted using forced entry. The correlation between the two variables in the Regression models were substantially below the “rule of thumb” threshold for multi collinearity (Dormann et al. 2013). IBM SPSS Statistics version 25 was used for computation.

## Results

### Narrative Ability Comparison Between Groups

Table 1 shows the age, group means, standard deviation and range for all language and narrative tasks for all three groups.

Comparing the BST measures, there were significant differences between the three groups: Information: H (119) = 28.60, *p* < 0.01; Sentence Length: H (119) = 56.13, *p* < 0.01; Subordinate Clauses: H (119) = 40.10, *p* < 0.01. Pairwise comparisons showed that the ASD group performed significantly poorer than the CAM group (*p* < 0.001*)* on all BST scores but at the same level as the LM group on BST Information (*p* = 1.0) (Fig. 2). Compared with the LM group, the ASD group performed significantly worse on BST Sentence Length (*p* < 0.05) and BST Subordinate Clauses (*p* < 0.05). As expected, the CAM and LM groups also differed significantly on all three BST scores, with the older children performing better: Information (*p* < 0.001), Subordinate Clauses (*p* < 0.05) and Sentence Length (*p ≤ *0.001).

Next, the NAP analyses showed significant group differences for all six dimensions: Topic Maintenance H [118 = 39.96, *p* < 0.001], Event Sequencing H [118 = 44.29, *p* < 0.001], Explicitness H [118 = 46.38, *p* < 0.001], Referencing H [118 = 23.5, *p* < 0.001], Conjunctive cohesion H [118 = 37.14, *p* < 0.001], Fluency H [118 = 57.86, *p* < 0.001], and NAP total H [118 = 52.50, *p* < 0.001]. For pairwise comparisons, see Table 2. Again, the ASD group differed significantly compared with both groups for all dimensions except Referencing, where no significant difference was found compared with the LM group. Comparing the CAM and LM groups, the older children performed better except that no group difference was found on Topic Maintenance, Event Sequencing or Fluency.

**Table 2:** Narrative discourse analysis (NAP) and group comparisons

NAP	Group comparison	*p* value
Topic maintenance	ASD < LM = CAM	ASD < CAM *p* < 0.001
		ASD < LM *p* = 0.001
		LM = CAM *p* = 1.0
Explicitness	ASD < LM < CAM	ASD < CAM *p* < 0.001
		ASD < LM *p* = 0.021
		LM < CAM *p* = 0.004
Event sequencing	ASD < LM = CAM	ASD < CAM *p* < 0.001
		ASD < LM *p* = 0.001
		LM = CAM, *p* = 0.16
Referencing	ASD = LM < CAM	ASD < CAM *p* < 0.001
		ASD = LM *p* = 0.982
		LM < CAM, *p* < 0.01
Conjunctive cohesion	ASD < LM < CAM	ASD < CAM *p* < 0.001
		ASD < LM *p* = 0.049
		LM < CAM *p* = 0.013
Fluency	ASD < LM = CAM	ASD < CAM *p* < 0.001
		ASD < LM *p* < 0.001
		LM = CAM *p* = 1.0
NAP total	ASD < LM < CAM	ASD < CAM* p* < 0.001
		ASD < LM *p* = 0.004
		LM < CAM *p* = 0.007

*ASD* autism spectrum disorder, *CAM* chronological age-matched group, *LM* matched on receptive and expressive language ability

#### Correlations Between Language, Narrating and Temporal Sequencing in the Group of Children with ASD

In order to understand the relationships between narrative ability and other language and cognitive variables, Spearman’s rho correlations were performed. These analyses were conducted in the ASD group only (since no temporal sequencing data had been collected for the comparison groups). As shown in Table 3, the results show strong correlations between all three BST scores, the NAP total and the language variables. There were also associations between BST scores and nonverbal temporal sequencing.

**Table 3:** Correlations between raw scores on narrative ability, assessed with the three sub-scores information, sentence length and subordinate clauses from the bus story test (BST), and raw scores on language and nonverbal cognitive variables

Variables	1	2	3	4	5	6	7	8	9
1. Age (years)	–								
2. Information (BST^a^)	.44**	–							
3. Sentence length (BST)	.51***	.78**	–						
4. Subordinate clauses (BST)	.41**	.81***	.81***	–					
5. Receptive grammar (TROG-2)	.55**	.70***	.61***	.60***	–				
6. Recalling sentences^b^ (CELF-4)	.35*	.71***	.57***	.59**	.64**	–			
7. Nonverbal cognitive ability	.39**	.43**	.49***	.40**	.57**	n.s	–		
8. Nonverbal temporal sequencing	.48***	.45**	.55***	.39**	.56**	.34*	.47***	–	
9. NAP total^c^	.48 **	.83***	.79***	.76***	.61***	.60***	.46**	.45**	–

Correlations were performed using Spearman’s Rho correlation

*n.s.* not significant

*p < .05, **p < .01, ***p < .001

^a^The bus story test

^b^Subtest CELF-4 total score

^c^Narrative discourse profile

#### Predictors of Narrative Skills in the Group of Children with ASD

The next step was to attempt to identify unique predictors of narrative performance using linear regression analysis. As dependent variables, the NAP total score and the three measures from the BST were included in separate analyses. Since autism symptomatology was not correlated with the outcome measures, it was not included in the regression models. The regression analyses were performed in two steps: first we entered age as a control variable, and in the subsequent step, two more explanatory variables were included, namely scores on the Recalling Sentences subtest as an index of expressive language at the sentence level and nonverbal temporal sequencing as per the Picture Arrangement Test. Together with age, these two independent variables contributed significantly to the regression model for Sentence Length (R^2^ = 0.49), for Subordinate Clauses (R^2^ = 0.33), for BST Information (R^2^ = 0.54) and for the NAP total score (R^2^ = 0.41), see Table 4. Recalling Sentences (i.e. expressive language) was a unique predictor for all BST outcome scores as well as for the NAP total score (*p* < 0.01). Nonverbal temporal sequencing was a unique predictor of Sentence Length (*p* = 0.003**),** whereas it fell shy below significance for the BST Information score (*p* = 0.06) and non-significant for Subordinate Clauses (*p* = 0.16) and NAP total (*p* = 0.13).

**Table 4:** Regression models with the dependent variables Information (BST), sentence length (BST), subordinate clauses (BST) and NAP total and the explanatory variables, recalling sentences and nonverbal temporal sequencing

	Information R^2^ = 0.54 F = 15.7 ***			Sentence Length R^2^ = 0.49 F = 12.7 ***			Subordinate Clauses R^2^ = 0.33 F = 6.5 **			NAP total R^2^ = 0.41 F = 9.4 ***		
	*β*	*t*	*p*	*β*	*t*	*p*	*β*	*t*	*p*	*β*	*t*	*p*
Age (constant)	0.13	1.0	0.32	0.15	1.1	0.26	0.19	1.3	0.21	0.12	0.88	0.38
Recalling sentences^a^	0.57	5.0	***	0.36	3.1	**	0.36	2.63	*	0.49	3.80	***
Nonverbal temporal sequencing^b^	0.24	1.9	0.06	0.41	*3.2*	**	0.21	1.41	0.17	0.21	*1.53*	0.13

^a^Recalling sentences from CELF-4, raw scores

^b^Picture arrangement, a WISC-III subtest, raw scores

*p < .05, **p < .01, *** < .001

## Discussion

The aim of this study was to better understand the nature of narrative difficulties in children with and without ASD, matched for age and language skills, respectively. Additionally, we aimed to examine the relation between narrative ability and nonverbal temporal sequencing by identifying explanatory variables for narrative ability in children with ASD using regression analyses. An important feature of this study is that we recruited participants from a population-based screening rather than from a pool of clinically referred cases; hence, the representativeness can be expected to be higher than in prior work on narrative performance in ASD.

Our first hypothesis was confirmed, i.e. the children with ASD performed worse than both chronological age-matched children and 2 years younger children matched on tests of expressive and receptive sentence-level language skills. Compared with the LM group, the ASD group exhibited significantly poorer performance on the BST, with their narratives containing shorter sentences and fewer subordinate clauses, suggesting that even though the groups were matched on language at the sentence level, the ASD group still produced less syntactically complex narratives. The only exception to this pattern was the BST Information score. When analysing the narratives further using the NAP analysis (Bliss et al. 1998), the group comparisons showed a similar pattern. Our children with ASD performed worse than the other two groups except for the Referencing score, where the ASD and the LM group performed at the same level. The Information score from BST and the Referencing score from the NAP likely capture similar abilities, since accurate referencing is important in order to achieve higher scores on BST Information. The NAP analysis comparison between the CAM and LM groups showed further that the younger and older children without ASD performed at similar levels on certain scores, namely Topic Maintenance, Event Sequencing, and Fluency, but the older outperformed the younger on Explicitness, Referencing, Conjunctive Cohesion and the NAP total. This pattern of results shows that several aspects of narrative development develop during the early school years in typically developing children.

The group comparisons revealed that language skills do not seem to fully account for narrative difficulties in ASD, as has indeed been suggested by results in some, but not all, previous studies. King et al. (2013) compared 12-year-old children with and without ASD matched on age, language skills and IQ, whereas Peristeri et al. (2017) compared three groups of 9-year-olds, ASD high language, ASD low language and non-ASD children, matched for language skills and IQ (Peristeri et al. 2017). Thus, just like we did, both of these studies found less syntactically complex narratives in children with ASD than in children without ASD. The results in the study by Peristeri et al. (2017), however, showed that the narratives produced by children with ASD and low language had significantly lower syntactic complexity (fewer subordinate clauses) than the other two groups, while there was no difference between ASD high language and the non-ASD children. In contrast, Diehl et al. (2006) found no differences in syntactic complexity between their ASD and a non-ASD group matched for age, gender, cognitive ability, receptive and expressive language, while there were significant differences in story coherence between the groups. Possibly, these differences between studies can be explained by the choice of test material for assessing narrating. Different test materials require different elicitation methods and potentially target different aspects of the narrative ability, such as story retelling or story generation and retention. The age of the child assessed and the representativeness of the study cohorts are other factors that may impact the results.

Our second hypothesis was only partly confirmed, since the role of nonverbal temporal sequencing did not consistently explain unique variance in narrative performance in the children with ASD. Hence, there seems to be an influence of temporal sequencing in oral narrative performance, but this seems to be contingent on the specific aspect of the narrative output considered. In the regression model, nonverbal temporal sequencing uniquely predicted individual differences in the Sentence Length BST subscore, and, at trend level, in the Information subscore (*p* = 0.06). Regarding the association to temporal sequencing, we previously argued on theoretical grounds that the Information subscore might mechanistically be more closely associated with temporal sequencing (Åsberg Johnels et al. 2013). But it is possible that the Information and Sentence Length subscores are both predicted by temporal sequencing since they collectively reflect comprehension of the original narrative and the ability to convey critical story elements in a syntactically efficient and correct manner (Renfrew 1997). In the future, these nature of the associations should be unravelled in greater detail, and attention should be given also to learning what specific abilities and functions are needed in order to solve the Picture Arrangement Task (Happé and Frith 2006; Language and Reading Research Consortium 2015; Marini et al. 2010; Zampini et al. 2017). In particular, an important task for the future would be to elucidate the associations between nonverbal temporal sequencing and other ASD-relevant cognitive skills, such as theory of mind, central coherence and executive functions (Happé and Frith 2006), and how these act as explaining factors for narrative performance.

Certain features of this study are potential weaknesses: First, we only assessed temporal sequencing in the ASD group and not in the two comparison groups; this choice was practical rather than theoretical and reflected time constraints at the schools. Another possible limitation of our study is that we used only one narrative cartoon task. The BST was chosen since it has been shown that retelling stories is suitable both for preschool children (Westerveld and Vidler 2015) and for older children with cognitive disabilities, as they on average produce longer and grammatically more complex narratives in story retelling narratives than in self-generated stories, where floor effects are common (Boudreau 2008; Merrit and Liles 1989). Interestingly, however, a recent review has in fact shown weak evidence of the common assumption that pictorial processing is a ‘strength’ in clinical populations, including autism (Coderre 2019). Instead, Coderre warns that more thorough consideration of the cognitive complexities in visual narrative processing is needed, and that it is not evident that narrative processing will become easier by merely adding visual stimuli (Coderre 2019). It could also depend on how the material is presented, verbally, in text or visually, and there might also be different patterns across development (Manfredi et al. 2020). An important avenue for future research is to examine how the choice of material and elicitation technique affects narrative performance in ASD and whether the predictors of narrative performance differ as a function of assessment method.

To summarize, in this study we have evidence for the hypothesis that children with ASD perform poorer on narrating than both younger language- and age-matched typically developing children. Moreover, we find partial evidence for our second hypothesis that, besides language skills, nonverbal temporal sequencing plays a role in narrative performance. Poor performance on this task might be taken as a proxy for weak central coherence (Happé and Frith 2006) or/and an index of a domain general deficit in narrative sequential reasoning (Coderre et al. 2018; Cohn 2014, 2019). It would be of interest to further investigate the skills and capacities that underlie nonverbal temporal sequencing and how they manifest in oral narration. Such insights could further theoretical development and have a potential impact on the design of interventions targeting narrative ability in children with ASD.
